# Impact of bowel dilation on small bowel motility measurements with cine-MRI: assessment of two quantification techniques

**DOI:** 10.1259/bjro.20210049

**Published:** 2022-02-21

**Authors:** Kyra L van Rijn, Jaap Stoker, Alex Menys, Catharina S de Jonge

**Affiliations:** ^1^ Department of Radiology and Nuclear Medicine, Amsterdam Gastroenterology, Endocrinology and Metabolism, Amsterdam UMC, University of Amsterdam, Amsterdam, The Netherlands; ^2^ Motilent Ltd., London, UK

## Abstract

**Objectives:**

To evaluate the effect of bowel dilation on cine-MRI small bowel motility measurements, by comparing a conventional motility score (including bowel wall and lumen) with a bowel wall-specific motility score in healthy and diseased populations.

**Methods:**

Four populations were included: 10 Crohn’s patients with a stricture and prestricture dilation for segmental motility analysis, and 14 mannitol-prepared healthy subjects, 15 fasted healthy subjects and eight chronic intestinal pseudo-obstruction (CIPO) patients (characterized by dilated bowel loops) for global small bowel motility analysis. All subjects underwent a cine-MRI scan from which two motility scores were calculated: a conventional score (including bowel wall and lumen) and a bowel wall-specific score. The difference between the two scores was calculated per population and compared between groups with a one-way ANOVA and Tukey-Kramer analysis.

**Results:**

In Crohn’s patients, the median (IQR) change between the conventional and wall-specific motility score was 0% (-2 to +4%) within the stricture and 0% (−1 to +7%) in the prestricture dilation. For the global small bowel, this was −1% (−5 to 0%) in mannitol-prepared healthy subjects, −2% (−6 to +2%) in fasted healthy subjects and +14% (+6 to+20%) in CIPO patients. The difference between the two motility scores in CIPO patients differed significantly from the four other groups (*p* = 0.002 to *p* < 0.001).

**Conclusions:**

The conventional small bowel motility score seems robust in Crohn’s disease patients and healthy subjects. In patients with globally and grossly dilated bowel loops, a bowel-wall specific motility score may give a better representation of small bowel motility.

**Advances in knowledge:**

These findings support researchers and clinicians with making informed choices for using cine-MRI motility analysis in different populations.

## Introduction

Quantified small bowel motility measurements using cine-MRI are increasingly used in clinical practice and published research, which reflects the interest for non-invasive techniques to assess gastrointestinal motility.^1,2^ To objectively measure gastrointestinal motility, several motility quantification techniques have been developed.^3–8^ One of these techniques ‘GIQuant^®^’ (Motilent, London, UK) uses pixel-based displacement measurements within a region of interest (ROI), such as the small bowel.^3,4^ Using this technique, a surrogate measure for small bowel motility is calculated within a ROI, which includes both the bowel wall and its’ luminal contents.

This technique has been used to assess small bowel motility in a broad range of populations,^4,9–14^ of which some have dilated small bowel loops. For example, patients with stricturing Crohn’s disease and a prestricture dilation (with reported diameters up to 10 cm^13^) or in the generalized dysmotility disorder chronic intestinal pseudo-obstruction (CIPO) where small bowel loops can be grossly dilated.^11–13^ In these populations the small bowel lumen may cover a large part of the ROI and may therefore have a disproportionate influence on the motility score (averaging down the motility score). Since research on assessment of small bowel motility with cine-MRI is increasingly performed for the purpose of clinical implementation, a robust and comparable quantification metric is of utter importance.

In patients with dilated bowel loops, a bowel wall-specific motility score might more accurately reflect small bowel motility than a score including both bowel wall and luminal content. A possibility to achieve this is by obtaining a motility score specifically for the bowel walls, for example by using an edge detection technique to detect bowel walls and use this as the ROI for motility calculations.

Comparing a conventional small bowel motility quantification technique with a small bowel wall-specific technique is an important step in the support of the development of a robust and reproducible quantification metric for cine-MRI motility assessment in research and clinic. This will allow us to choose the appropriate motility quantification technique (or approach) for different populations and enables us to adequately interpret results from previous and future studies.

The aim of this study is to evaluate the effect of bowel dilation and luminal content on small bowel motility measurements, by comparing conventional quantified small bowel motility measurements including luminal content with a new wall-specific motility assessment workflow using edge detection in healthy and diseased populations.

## Methods and materials

### Ethical

Data were collected from three prospective studies which were approved by the local institutional review board and for which patients had signed informed consent.^10,12^

### Population

**Figure 1.**
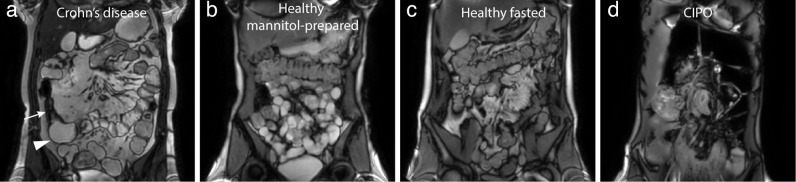
MRI examples of the included populations with different small bowel characteristics. (a) shows a Crohn’s disease patient with a stricture (tailed arrow) and prestricture dilation (arrowhead), (b) a mannitol-prepared healthy subject, (c) a healthy fasted subject and D) a CIPO patient with dilated small bowel loops.

Four populations with different small bowel characteristics were included to evaluate the effect of bowel dilation and luminal content on small bowel motility measurements. Table 1 shows the characteristics of the included populations. Figure 1 shows an example of an MRI scan per population. Collectively these subjects expose the motility quantification algorithm to some very mixed cases. As we increasingly use MRI for radiologically poorly characterised disease, we feel this is a robust way of assessing the research question.

**Table 1.** Characteristics of the included populations

Population	N=	Global or segmental motility analysis	Small bowel preparation	Small bowel characteristics
Crohn’s patients with a stricture and prestricture dilation	10	Segmental (stricture and prestricture dilation ROI)	Mannitol, 1.6 L of 2.5%	Strictured bowel segment with an upstream prestricture dilated bowel loop
Mannitol-prepared healthy subjects^10^	14	Global	Mannitol, 1 L of 2.5%	Normal calibre, fluid-distended small bowel loops
Fasted healthy subjects^10^	15	Global	None, fasted overnight	Normal calibre, variably/somewhat sparsely filled small bowel loops
CIPO patients^12^	8	Global	None, fasted overnight	Various, mixed small bowel configurations, both normal calibre and severely dilated small bowel loops

### MRI protocol

All subjects underwent cine-MRI in supine position using a 3T scanner (Philips Ingenia, Best, the Netherlands) with an anterior torso-coil covering the entire abdomen and a posterior coil located in the table. For small bowel motility analysis, a dynamic balanced Fast Field Echo (bFFE) sequence was acquired within a 20 s breath-hold (BH). The scan parameters of the different populations are shown in Table 2.

**Table 2.** Scan parameters of the included populations

Population	Cine-MRI scan	TE (ms)	TR (ms)	Flip angle	FOV (mm)	Spatial resolution (mm)	Temporal resolution (fps)
Crohn’s patients	bFFE	1.26	2.5	20°	400 × 400 x 35	2.5 × 2.5 x 2.5	1
Healthy subjects	bFFE	0.89	1.90	20°	400 × 400	2.5 × 2.5 x 10	2 (undersampled from 10)
CIPO patients	bFFE	1.13	2.27	20°	400 × 400	1.8.×1.8 x 10	2

FOV, Field of view; TE, echo time; TR, repetition time; bFFE, balanced fast field echo; fps, frames per second.

For every subject, a single 2D slice was used for motility analysis. In Crohn’s patients, scans were acquired in a 3D volume; a 2D slice visualising the dilation and a 2D slice visualising the stricture were extracted from this volume and used for motility analysis. In healthy volunteers and CIPO patients the 2D slice location was determined during scanning by an experienced MRI motility researcher. The slice was positioned at a location containing the greater part of the small bowel. Scans from healthy subjects were undersampled to two fps before motility analysis was performed since this frame rate is more suitable for use in clinical practice^15^ and this frame rate is comparable with the CIPO population.

### Small bowel motility analysis

#### Cine-MRI registration and quantification

Cine-MRI scans were analysed with a validated and CE-marked post processing tool (GIQuant^®^, Motilent, London, UK).^3,4^ The GIQuant^®^ algorithm is based on non-rigid registration and measures displacement per pixel using the standard deviation (SD) of the Jacobian. This technique results in a parametric motility map. Lower scores represent lower deformation in the time-series data and therefore lower motility and vice versa.

#### Delineation ROI

**Figure 2.**
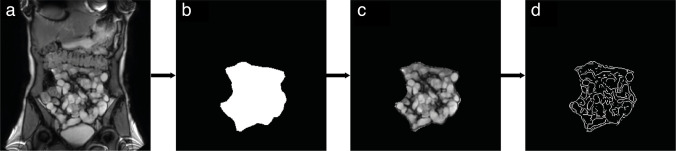
Delineation of the global small bowel in a mannitol-prepared healthy subject. (a) shows the reference image on which the ROI was delineated, resulting in b) a binary mask of the small bowel. (c) shows the remaining reference scan after the binary mask (b) was applied, resulting in a reference image with small bowel only. On this image, edge detection was applied resulting in d) the wall-specific binary mask.

Motility data visualization and motility quantification was performed in MATLAB 2019b (The MathWorks, Natick, MA, USA). To measure small bowel motility, first a ROI was manually drawn around the small bowel of interest for each subject in the static reference image of the cine-MRI scan (Figure 2a). Within this ROI, a binary mask (Figure 2b) was created for further calculations. In Crohn’s patients, two segmental ROIs were delineated: one including the stricture and one including the prestricture dilation. These segmental ROIs were delineated because they represent both narrowed and dilated bowel loops and are relevant in the evaluation of Crohn’s disease.^13^ In the healthy and CIPO populations, the ROIs consisted of the entire small bowel visible in one slice for global assessment of small bowel motility.

#### Bowel wall-specific ROI

To create a wall-specific ROI, the bowel walls were detected within the segmental and global ROIs (1C) with an edge detection method using a Sobel operator in MATLAB R2019b (The MathWorks, Natick, MA, USA). Several edge detection methods and settings were tested in subjects of the studied populations and the visual correspondence of the edge detection masks with the bowel walls was discussed by the study team. We scrutinised the algorithm to the point where we, as experienced readers, could not see improvements in detection of the bowel walls. This assessment resulted in the selection of the Sobel edge detection operator since it corresponded best with the bowel walls on the MRI scans. The Sobel edge detector uses a 3 × 3 convolution kernel to detect changes in gradients in an image; a sharp change in signal intensity in the image is detected as an edge. Running the edge detection operator resulted in a bowel wall-specific binary mask (Figure 2d). The percentage drop of included number of pixels in the wall-specific mask versus the conventional mask was calculated per group.

#### Motility score calculation

**Figure 3.**
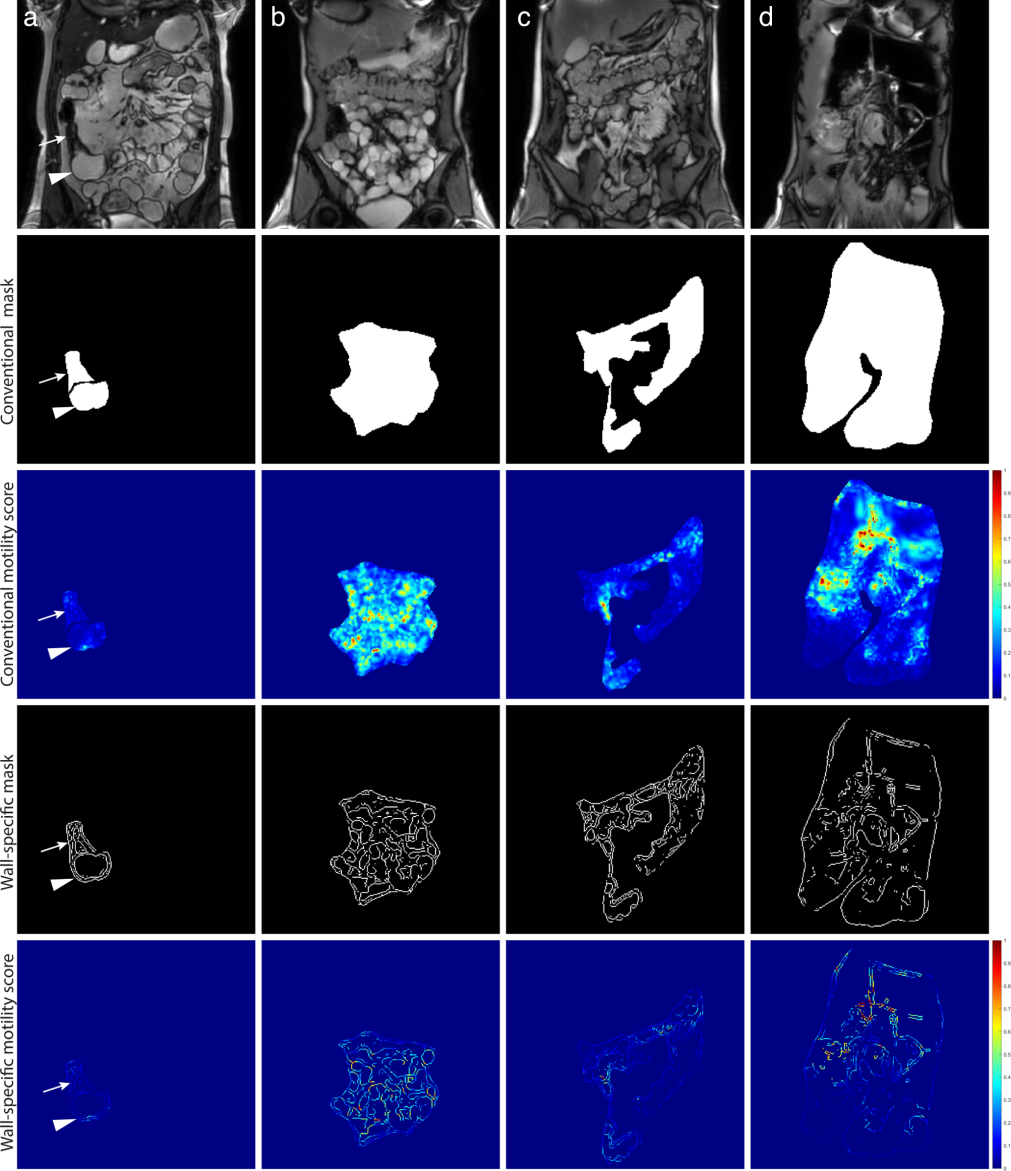
MRI examples, binary masks and motility maps of the four included populations. The first row shows the reference MRI scans of the different populations, (a) shows a Crohn’s disease patient with stricture (tailed arrow) and prestricture dilation (arrowhead), (b) a mannitol-prepared healthy subject, (c) a fasted healthy subject and d) a CIPO patient with dilated bowel loops. The second row shows the conventional binary masks and the third row the corresponding motility maps from which the conventional motility score was calculated. The fourth row shows the wall-specific binary masks and the fifth row show the motility maps from which the wall-specific motility scores were calculated. For the Crohn’s patients, a motility score for the stricture and motility score for the prestricture dilation were calculated. Areas of high motility are shown in red and low motility in blue.

To calculate the motility scores for both the conventional method and for the wall-specific method, the two different binary masks (*i.e.,* binary mask of the small bowel (bowel wall and lumen) and small bowel wall-specific binary mask) were applied to the motility map derived from the GIQuant^®^ algorithm. This resulted in two different motility maps, one including the overall ROI and one including the wall-specific ROI. Figure 3 shows an example of the two binary masks and the two motility maps for the different populations. From the motility map including small bowel wall and lumen, a conventional motility score was calculated by averaging all pixels. From the edge detection motility map a motility score specific for the bowel wall only was calculated. Both motility scores were calculated for every subject, all motility scores were presented in arbitrary units (AU).^3^

### Visual assessment

Cine-MRI scans were visually assessed by an abdominal radiologist (JS,>25 years’ experience). Small bowel motility was visually assessed on a 3-point scale (static/sparsely motile – motile – very motile).

For global small bowel motility the following definitions were stated: static/sparsely motile was defined as >50% of the small bowel loops being static and an absence of very motile small bowel loops. Very motile was defined as >50% of the small bowel loops being very motile. The rest was classified as motile.

Small bowel calibre was measured in mm in one measurement in Crohn’s disease and in four quadrants in the global populations of which the mean was calculated afterwards. Also, the predominant luminal content, either gas or fluid (>50%), was visually assessed.

The strictured segment in Crohn’s disease was not assessed for luminal diameter and luminal content as we deemed this irrelevant in a narrowed bowel loop.

### Statistical analysis

The difference between the conventional and wall-specific motility score was calculated in percentage ((wall-specific –conventional)/conventional*100) per ROI. The difference between the two motility scores expressed in percentage was compared between the five types of ROIs with a one-way ANOVA and post hoc Tukey Kramer analysis.

Compared to existing literature^4^ with the GIQuant motility score, the CE-Marked GIQuant^®^ tool presents motility scores times 1000 for the purposes of clear presentation in the clinical setting. In this study all values were converted back, dividing them by 1000, resulting in a score between 0 and 1, so that they may be interpreted within the context of the literature. Motility scores were presented as medians with interquartile ranges (IQR).

## Results

### Small bowel motility scores

Crohn patients (segmental scores): The median (IQR) motility score within the strictured segment was 0.16 (0.13–0.21) with the conventional technique and 0.16 (0.12–0.22) using the wall-specific technique. For the pre-stricture dilation, this was 0.21 (0.19–0.29) and 0.21 (0.18–0.28), respectively.

Healthy subjects (global scores): In mannitol-prepared healthy subjects the conventional motility score had a median (IQR) of 0.31 AU (0.27–0.32) and the wall-specific motility score 0.29 (0.27–0.32). In fasted healthy subjects, this was 0.14 (0.12–0.17) and 0.14 (0.13–0.17), respectively.

CIPO patients (global scores): In CIPO patients the conventional motility score was 0.17 (0.14–0.24) and the wall-specific motility score was 0.20 (0.15–0.29).

### Difference between the two techniques

**Figure 4.**
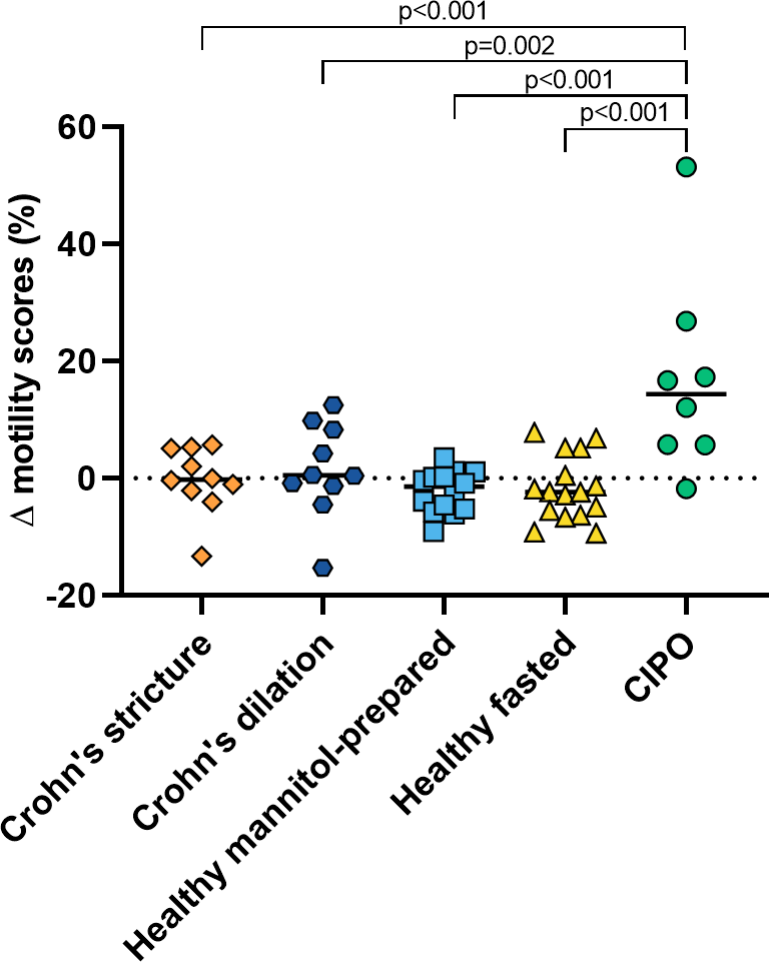
Percentage difference (δ) between the conventional and wall-specific motility score per population; an increase shows that the wall-specific motility score is higher than the conventional motility score.

Figure 4 shows the differences between the two motility scores per population. In Crohn’s patients, the median change between conventional and wall-specific motility score was 0% (-2 to +4%) within the stricture and 0% (-1 to +7%) for the prestricture dilation. For global small bowel motility, the median change in motility score was −1% (-5 to 0%) in mannitol-prepared healthy subjects, −2% (−6 to +2%) in healthy fasted subjects and +14% (+6 to+20%) in CIPO patients.

One-way ANOVA showed a significant difference between the five groups (*p* < 0.001*) and Tukey-Kramer post hoc analysis showed that the difference between the two motility scores in CIPO patients was significantly different from all other groups (*p*-values ranging from 0.002 to <0.001).

Table 3 shows the percentage drop of included number of pixels in the wall-specific mask compared to the conventional mask.

**Table 3.** Percentage drop of included number of pixels in wall-specific mask versus conventional mask

Population	Median drop of pixels in percentage
Crohn’s dilation	−72%
Crohn’s stricture	−62%
CIPO	−77%
Healthy prepared	−78%
Healthy unprepared	−75%

### Visual analysis

**Figure 5.**
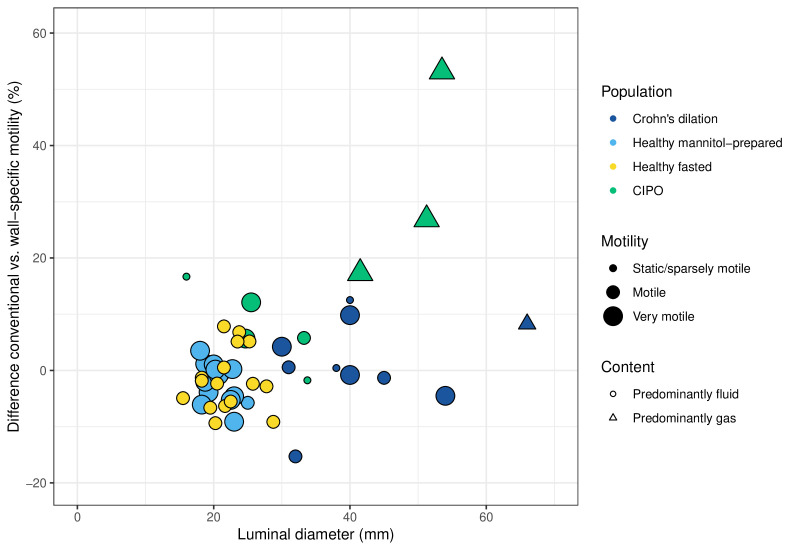
Luminal diameter in relation to the difference between the conventional and wall-specific motility score; an increase shows that the wall-specific motility score is higher than the conventional motility score. The populations are shown in colours, the luminal content in shapes and motility in sizes (luminal diameter was not assessed in the Crohn’s stricture population therefore this group is not shown).

Figure 5 shows the visually assessed motility, luminal diameter and luminal content in relation to the difference between the conventional and wall-specific motility score. The three CIPO patients with the largest bowel diameter and gas-filled bowels showed the most difference between the two motility scores, these three had very motile small bowel loops.

## Discussion

In this study, we demonstrated that motility measurements, using a pixel-based displacement mapping technique including luminal content, are robust in Crohn’s patients and healthy subjects. However, in CIPO patients with globally dilated bowel loops a wall-specific motility measurement technique seems preferable.

Today, cine-MRI motility measurements are clinically used in several centres to provide reassurances on the localisation of disease activity in small bowel Crohn’s disease. In research, MRI motility measurements are increasingly used to explore bowel physiology in a range of healthy and diseased subjects.^1,10,16–19^ The ability to now quantify small bowel motility is exciting but remains at an early stage. As these techniques are likely to become more widespread, especially now with commercially available analysis technologies, it Is crucial that we investigate how we use these tools, tackling limitations and exploring emerging needs and questions. Here, the effect of dilated bowel, a hallmark of a range of dysmotility disorders, on small bowel motility measurements was investigated to gain insight into the robustness of these measurements in different populations. We split this discussion across the clinical and research domains.

Important for clinical use, our results show that both a quantification technique including bowel walls and luminal content and a bowel wall-specific technique are robust for measuring small bowel motility in stricturing Crohn’s disease. In the strictured small bowel loops and even in the dilated bowel loops, there was no clear difference between the two motility scores. These findings are reassuring, as most of the previously published clinical work on MRI motility measurements was focussed on Crohn’s disease and introducing an extra analysis step in clinic is possible but complex and therefore undesirable. Additionally, it would also limit interpretation of previous studies.^9,13,14,20–22^ Our results show that the workflow as it is, using the conventional technique that includes luminal content, is reliable for clinical use in Crohn’s disease patients.

For research, there are many applications in which there is an interest in measurement of small bowel motility using MRI, which one day may be used in clinical practice.^11,12,16,17^ In this study, we included CIPO patients, a population with an extremely abnormal bowel configuration.^11,12,16^ In these patients, the bowel-wall specific motility score was higher than the conventional motility score, in contrast to the healthy and Crohn’s populations. The largest difference between the two motility scores was seen in patients with gas-filled, dilated bowel loops, these patients had visually very motile bowel loops. This finding indicates that the luminal content in globally dilated small bowel loops has a considerable impact on the motility calculations. Also, the similar percentage drop of included number of pixels in the two binary masks between the global populations indicates that the motility score is mainly affected by which pixels are excluded from the analysis (pixels representing gas-filled lumen) and not the number of pixels. In cases with dilated bowel loops, a wall-specific motility score may be well needed as we risk biasing down the motility scores with the conventional technique. When it comes to determining cut-off scores of normal versus diseased small bowel motility in the future, this might be an important consideration. In healthy populations, the motility quantification tool was robust, indicating that there is no need to include an extra analysis step for studies in this population.

This study was focused on the GIQuant motility metric available through Motilent,^3^ however considering the technical basis to calculate the motility score, it can be expected that the results are translatable to other bowel motility metrics that are built on similar principals measuring deformation or pixel intensities.^3^

In this study, we chose the edge detection method that visually corresponded best with the detection of the bowel walls. However, to truly validate an edge detection technique, a more technical approach should be applied based on mathematical concepts. We believe that for the aim of this study the chosen method was sufficient, which was also supported by the robust measurements in the healthy and Crohn’s populations. To avoid an extra analysis step with an edge detection technique, a workaround of manually delineating the small bowel walls can serve as another approach to analyse bowel walls without luminal content in subjects with dilated small bowel loops. However, this might be more time consuming than using the edge detection technique, especially for global small bowel motility measurements.

Limitations of this study are the population sizes; the population with gas-filled dilated bowel loops was small, the effect however was substantially clear in these patients. The healthy and Crohn’s populations were adequately large to show that there was no effect of the wall-specific motility score. However, to draw firm conclusions and to investigate the magnitude of the effect of dilation on conventional small bowel motility measurements, investigation of larger groups with dilated bowel loops is necessary. Furthermore, patients with globally dilated, fluid-filled bowels were underrepresented in this study making it difficult to establish the effect of luminal content aside from dilation. However, one can argue the relevance of this group since in current daily practice, subjects with this feature rarely are referred for MRI of the small bowel. Healthy volunteers prepared with mannitol might be closest to a fluid-distended global small bowel and this group showed no difference between the two motility scores. Additionally, in Crohn’s patients the dilated segment was mostly filled with fluid and in this population the motility scores were similar as well. Also, in this study we visually assessed motility, luminal diameter and content to evaluate the influence of these factors on the conventional motility score. An experienced abdominal radiologist reviewed these aspects, however visual assessment of motility remains difficult and has shown poor interobserver agreement in Crohn’s patients^23^ making it an imperfect reference standard.

In conclusion, this study shows that conventional small bowel motility measurements based on displacement mapping are robust in stricturing Crohn’s disease patients and in healthy subjects with normal calibre-bowel. For subjects with grossly dilated bowel loops, a wall-specific motility score is preferred over a quantification technique that includes luminal content. Our findings support informed choices for cine-MRI motility analysis in clinic and research.
